# Leg ulcers in sickle cell disease patients

**DOI:** 10.1590/1677-5449.200054

**Published:** 2020-11-11

**Authors:** Paula Dadalti Granja, Samuel Braulio Magalhães Quintão, Franciele Perondi, Rosemary Bacellar Ferreira de Lima, Cláudia Labriola de Medeiros Martins, Marcos Arêas Marques, Julio Cesar Peclat de Oliveira

**Affiliations:** 1 Departamento de Medicina Clínica – MMC, Faculdade de Medicina, Universidade Federal Fluminense – UFF, Niterói, RJ, Brasil.; 2 Hospital Universitário Antônio Pedro – HUAP, Niterói, RJ, Brasil.; 3 Hospital Universitário Pedro Ernesto, Universidade do Estado do Rio de Janeiro – UERJ, Rio de Janeiro, RJ, Brasil.; 4 Hospital Universitário Gaffrée e Guinle, Universidade Federal do Estado do Rio de Janeiro – UNIRIO, Rio de Janeiro, RJ, Brasil.; 5 Cirurgia Vascular, Universidade Federal do Rio de Janeiro – UFRJ, Rio de Janeiro, RJ, Brasil.

**Keywords:** sickle cell, leg ulcers, wounds, hemoglobinopathy, complications

## Abstract

Leg ulcers are the most common cutaneous complication of sickle cell disease. These lesions occur mainly in homozygous forms, are slow to heal and often relapse, causing negative physical, emotional, and economic impacts. In this paper, we discuss the clinical presentation, diagnosis, and pathophysiology of sickle cell leg ulcers and their implications for treatment.

## INTRODUCTION

Sickle-cell disease (SCD) is an autosomal recessive genetic disorder that affects the shape and function of red blood cells, causing a series of systemic complications. A mutation on chromosome 11 that codes the hemoglobin beta chain results in pathological polymerization of this protein.^1^^,^^2^ As a consequence, some red blood cells become denser and lose their capacity to deform and flow through the microvascularization, causing vasoocclusion, inflammation, ischemia, tissue damage, and hemolytic anemia.^1^^,^^2^ The vascular disease component of SCD can provoke pulmonary hypertension, pain crises, hand-foot syndrome, ischemic stroke, priapism, and lower limb ulcers.^1^

Up until the 1960s, SCD caused high mortality rates in young patients, with a mean age of ten years. Medical developments such as early diagnosis with neonatal screening (the Guthrie test), systematic immunization, prophylactic antibiotic therapy for the five first years of life, systematization of use of hemocomponents and iron chelators, hydroxyurea therapy, and screening with transcranial Doppler for primary prevention of strokes have changed the natural history of SCD patients.^3^ Nowadays, the majority of patients reach adulthood and, as a result, the complications of the disease have become more common.^4^

Leg ulcers are the most common cutaneous manifestations of SCD^5^ and can be incapacitating.^1^ They affect young patients, from the second decade of life onwards; cause intense, chronic, and continuous pain, and have a high rate of relapse. These ulcers have a significant impact on quality of life, can cause depression, and are responsible for a considerable increase in healthcare costs.^2^^,^^6^

## METHODOLOGY

The format of this study is a non-systematic review of the literature indexed on the PubMed database, using the keywords: *“Sickle cell” and “ulcers”* and *“Sickle cell” and “wounds”*. Meta-analyses and review articles published in the last 5 years, random allocation studies, and case reports were included. Additionally, the reference lists of the articles selected were also searched.

## EPIDEMIOLOGY

It is estimated that approximately 3,000 new cases of SCD emerge every year in Brazil.^7^ Leg ulcers are ten times more common among people with SCD than in the general population and SCD is therefore considered a powerful risk factor for skin ulceration.^4^

The prevalence of ulcers among SCD patients varies geographically, with rates that range from 75% in Jamaica to 1% in Saudi Arabia.^5^ In Brazil, the prevalence of these ulcers is 20% of SCD cases.^3^

Data from Jamaica indicate that leg ulcers are rare before ten years of age, with onset most frequently from 10 to 25 years of age. The United States’ Cooperative Study of Sickle Cell Disease has estimated that the prevalence of ulcers varies from 5 to 10% and that maximum incidence is between 20 and 50 years of age.^8^

## PATHOPHYSIOLOGY

It is essential to understand the underlying pathophysiologic mechanisms to choose a successful treatment approach. The pathogenesis of leg ulcers in SCD ha not been completely elucidated and appears to be multifactorial. After the oxygen levels in tissues fall, hemoglobin S molecules polymerize and stick together, distorting the membranes of red globules, resulting in red blood cells with the typical sickle shape. Intravascular precipitation of the red blood cells results in vasoocclusion, endothelial dysfunction, hypercoagulability, chronic inflammation, and ischemic tissue damage.^5^

Intravascular hemolysis occurs, allowing free hemoglobin to sequestrate nitric oxide (NO), reducing its vasodilator action on the vascular endothelium, intensifying the chronic vasoconstriction, hypoxia, and pain.^5^ Recent studies have investigated the role of NO in SCD, observing reduced NO levels during complications.^9^

The literature emphasizes the role played by chronic venous disease (CVD) in the pathogenesis of leg ulcers in SCD.^5^ The fact that there is no evidence that SCD patients have problems with healing in areas other than the legs suggests that specific local factors are related to the pathophysiology of these ulcers.^8^ Studies of changes in the volume of blood in the ankle during physical exercise show that venous filling times are shorter in people with SCD compared with controls without the disease; and times were even shorter in patients who had sickle-cell ulcers compared to those who did not.^8^ This confirmed the following hypothesis: insufficiency of the venous valves that drain the region of the ankle and constantly elevated venous pressure contribute to slow healing and possible onset of leg ulcers in SCD.^8^ Jamaican cohort studies conducted with portable Doppler ultrasound found CVD in 75% of 183 people with SCD compared with 39% of 137 controls without SCD, demonstrating a significant association (p < 0.001).^8^

Histopathological investigations found evidence of microthrombi and fibrin deposits in the lumen of blood vessels from both the ulcers and from areas around them, suggesting pathological hemostasis.^10^ Obstruction of microvessels, induced by sickling of red blood cells, stimulates expression of vascular endothelium adhesion elements, platelet aggregation, and release of proinflammatory cytokines, exacerbating the obstruction, ischemia, and necrosis.^5^ Recently, Brazilian researchers have detected elevated levels of Interleukin-8 in patients with SCD and leg ulcers, emphasizing the role of inflammation in the pathogenesis of these lesions and suggesting that this cytokine can be considered a marker of poor prognosis.^11^ Autonomic dysfunction also occurs. Venous-capillary reflex responses are aberrant and cutaneous vasoconstriction is more pronounced in the ulcer site when the leg is lowered.^5^

The combination of microvascular occlusion, inflammation, and thrombosis increases the risk of a patient developing ischemia. The consequent tissue damage provokes cyclic events (valve damage, for example) which aggravate the tissue damage even further and increase fluid retention and inflammation, creating an environment favorable to ulceration.^5^

## CLINICAL CHARACTERISTICS OF ULCERS

According to Serjeant et al.,^8^ SCD leg ulcers can have traumatic or spontaneous onset. Traumatic injuries account for half of cases, while spontaneous cases begin in the dermis. Pain at the site of the lesion is one of the most characteristic manifestations of sickle-cell ulcers, which have a punched out appearance with well-defined limits and raised edges, as is also observed with ischemic ulcers. The base is lined with granulation tissue, sometimes covered by yellowed slough. Sometimes, several small ulcers appear simultaneously and coalesce, forming one larger ulcer. Edema of the extremity involved is a common finding. The skin around the ulceration may be hypopigmented or hyperpigmented, indicating previous injuries, hair follicles are sparse and the musculature is somewhat atrophied.

Generally, ulcers affect the skin around the medial or lateral malleoli, which are areas that are more vulnerable to mechanical vascular obstructions, with the result that minor abrasions become foci of inflammation, ischemia, and tissue damage.^5^ Additionally, physical examination generally reveals the stigmata of CVD, such as cutaneous hemosiderosis, dermatosclerosis, and prominent superficial veins. Additional evidence is the tendency for these ulcers to worsen in orthostasis and improve with rest and with compression therapy.^5^

Cure is slow and takes months or years. Secondary bacterial infections are almost inevitable and the most common etiologic agents are *Staphylococcus aureus*, *Pseudomonas aeruginosa*, and *Streptococcus pyogenes.* Finally, scarring results in low resistance to traction and the poor perfusion due to vascular cutaneous disease cause an increased propensity to wound reopening.^4^

## CLASSIFICATION

In 2016, Minniti et al.^4^ proposed three patterns of leg ulcers in patients with sickle-cell disease (SCD). The description found support from other scholars of the subject and was used in later publications.^2^

## SINGLE ULCERS, RECURRENT ULCERS, AND CHRONIC RECURRENT ULCERS

The single ulcer occurs in patients who develop only one ulcer over the course of their lives, healing in a few months. They typically occur in the second decade of life and may recur in periods of stress. These patients have a low frequency of pain crises and may have pulmonary or renal complications.

The recurrent type ulcers are small and recur every 6 to 12 months for several years. They are observed in one quarter of patients and tend to respond better to treatment. Although recurrence is a cause of considerable concern for these patients, they don’t generally tend to be severely debilitated by their ulcers.

Finally, chronic recurrent ulcers are lesions that last for years or recur in the same areas or close by. This presentation causes more debilitating and chronic pain and the patient suffers depression, incapacity, and unemployment. Although ulcers heal in 75 to 80% of cases, many patients can have ulcers that last for more than 20 years or never heal. In some cases, amputation of the limb may be considered to improve quality of life.

## DIAGNOSIS

Although the majority of patients with SCD are diagnosed early at large centers, we should be alert to the possibility in young patients with leg ulcers, because SCD can sometimes be diagnosed late as a result of complications.

A detailed history should be taken, covering information on current treatments and complications of previous treatment. Physical examination should assess hypopigmentation or hyperpigmentation around the lesion, limb edema, inguinal lymph nodes, and the size of the ulcer. We should also observe the characteristics and volume of exudate and the appearance of the wound bed. Differential diagnosis of ulcers in SCD patients includes the many causes of chronic leg ulcers, such as vascular ulcers (arterial, venous, and mixed), hypertensive ulcer (Martorell’s ulcer), infectious ulcers (in Brazil, leishmaniasis, sporotrichosis, and micobacterioses are the most common), medication-induced ulcers (hydroxyurea and methotrexate, for example), ulcers related to neoplasms (such as basal cell carcinoma, squamous cell carcinoma, melanoma, and cutaneous metastases), ulcers related to autoimmune diseases (systemic sclerosis, systemic lupus erythematosus, and rheumatoid arthritis), and primary cutaneous conditions (necrobiosis lipoidica, sarcoidosis, and pyoderma gangrenosum, for example).^12^ Laboratory diagnosis of SCD is based on identification of hemoglobin S (HBS) or other variants of hemoglobin and should be performed by electrophoresis of hemoglobin.

Laboratory tests such as urinalysis, complete blood count, and biochemistry should be ordered. Microalbuminuria and markers of severe chronic hemolysis may be observed, facilitating diagnosis.^2^

Histopathological analysis of ulcer biopsy specimens may show atrophic epithelial borders, increased vascularity, vascular disease with vascular occlusion, chronic inflammatory process, microthrombi, and intimal fibrin deposits.^2^ Although these findings are not very specific, they are particularly useful for differential diagnosis from other causes of lower limb ulcers.

## TREATMENT

Treatment of leg ulcers in patients with SCD should take a holistic approach to reduce the physical and psychological impacts of a chronic illness, with a slow healing process, and high rates of recurrence. However, guidelines based on scientific evidence to direct appropriate management of treatment are lacking. Health professionals therefore base their conduct on critical literature reviews and on personal and clinical experience when treating these ulcers.^4^

## GENERAL CARE

In patients with ulcers, compliance with treatment can be considered the principal determinant factor of successful healing.^5^ A multidisciplinary approach is essential, closely monitored by hematologist, vascular surgeon, angiologist, dermatologist, nurse, nutritionist, and psychologist.

From a nutritional point of view, we should be alert to the possibility of zinc deficiency, since correction can help with healing. The current recommendation is 220 mg of zinc sulfate three times a day, with reassessments after 3 to 4 weeks and withdrawal if the reference levels have been achieved.^5^ Testing for and treatment of superficial and/or deep venous thrombosis is fundamental, possibly requiring anticoagulation.

Pain management should be a priority. Because of the adverse effects profile of chronic use of opioids, non-steroidal anti-inflammatories are generally recommended. On the basis of personal observations, Altman et al. suggest regional nerve block, with the advantage of secondary vasodilation by reducing release of catecholamines by stress.^5^ On the other hand, compliance may be compromised by the need for an invasive procedure, the risk of infection of soft tissues, and the need for frequent visits for readministration.

## LOCAL CARE

Wound healing is a complex and dynamic process that can be simplified in three phases. In the first, inflammatory phase, there is leakage of blood and platelets release growth factors that attract and activate fibroblasts, macrophages, and leukocytes. During this phase, therapeutic focus should be on debridement and infection control. The majority of chronic ulcers are in this phase. The longer the ulcer remains in the inflammatory phase, the less deposition of collagen there will be, resulting in lower resistance to traction in the new skin. In the second, proliferative phase, epithelialization begins, granulation tissue is formed and the wound begins to contract. In the final phase, maturation and remodeling, collagen break-down is controlled by proteolytic enzymes.^4^

The basic principles of wound treatment are didactically simplified by the mnemonic TIME: Tissue debridement; Infection/inflammation control; Moisture balance; and Edge of wound epithelialization.^13^ Treatment of the ulcer bed is essential to the healing process. Debridement of biofilm, fibrin, and non-viable necrotic tissue from the base and edges is essential and is considered the first step to remove physical barriers that interfere with healing.^14^ The method (autolytic, enzymatic, biological, mechanical, or surgical) will be chosen on the basis of the type of wound, anatomic location, and size, and on availability. Surgical debridement may require analgesia or even anesthesia because of the pain involved.

Control of bacterial colonization is important to the choice of covering, and some substances, such as silver, iodine, and polyhexamethylene biguanide, have antibacterial properties and can reduce critical colonization.^13^^,^^15^ There is no evidence that treating clinically uninfected ulcers with systemic antibiotics prevents infection or improves healing. When there are clinical signs of infection, a deep sample of soft tissue or bone can be collected after debridement, for culturing and assessment of sensitivity to antimicrobials.

Maintenance of a humid environment can be achieved by choosing an appropriate covering, such as hydrocolloids, hydrogels, alginates, collagen, or biological skin substitutes. Some of these coverings also have anti-inflammatory and autolytic debridement properties.

A matrix of peptides, known as RDG, was mentioned in a Cochrane review (2014) as effective for reducing the size of treated ulcers, compared with placebo.^16^ However, the evidence is not very consistent. Other studies analyzed in the meta-analysis had a high risk of bias and failed to demonstrate beneficial effects.^17^

New technologies, such as low-intensity lasers, have been used with the objective of accelerating healing and thus improving patients’ quality of life^5^^,^^18^ (evidence level 4, case report). One fundamental effect is the capacity to reduce pain, enabling a more effective approach when applying dressings.

## TREATMENT OF CVD

**Figure 1:**
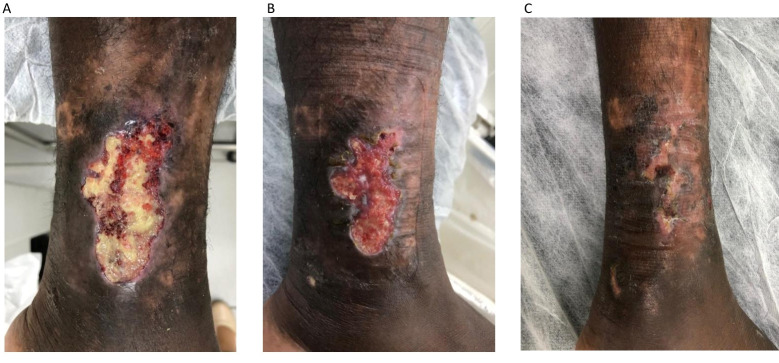
A 39-year-old patient diagnosed with sickle-cell disease in childhood. The images show: (A) an ulcer measuring around 8 cm at its largest diameter, with the majority of the bed covered by fibrin; (B) management with polyaminopropyl biguanide followed by application of a sodium carboxymethyl cellulose and silver covering in addition to weekly Unna boot; and (C) the patient achieved complete healing after 2 months of treatment.

Compression therapy is encouraged for prevention and treatment of edema, especially when clinical signs of CVD are observed. Graduated elastic compression stockings are useful for prevention, while multilayered bandaging is recommended for treatment. Another option is self-administered and self-adjusted bandages with velcro, but they are more subject to errors of application.^5^ The recommendation level for compression to accelerate healing in patients with CVD is 1 (evidence level A) and for reducing the risk of recurrence, the recommendation level is 2 (evidence level B).^5^ We illustrate the response to compression therapy with an Unna boot in a patient receiving care at our clinic (Figure 1).

Pentoxifylline is a methylxanthine derivative that is a competitive phosphodiesterase inhibitor that has shown antioxidants and anti-inflammatory properties. It also reduces the viscosity of the blood and its potential for platelet aggregation and clot formation.^19^ Its use in sickle-cell ulcers had already been proposed in 1990, on the basis of its effects in reducing sickling of red blood cells in vitro and for resolution of vessel-occlusive crises in SCD (evidence level C, recommendation level 4).^20^

This drug has been strongly recommended for treatment of venous stasis ulcers (evidence level B, recommendation level 1) and some authors consider that it should also be used in patients with sickle cell ulcers because of the high frequency of CVD observed in these cases.^5^ The same group^5^ also suggests using a biological skin substitute (Apligraf®, Organogenesis Inc., Canton, Massachusetts), composed of cultured keratinocytes and fibroblasts on a collagen scaffold, for treatment of leg ulcers in patients with SCD and CVD, who have not responded to conventional treatments after 4 to 6 weeks. In the case of CVD ulcers, the evidence level is A, recommendation level 2.^5^

## HYDROXYUREA

Hydroxyurea is widely used for treatment of SCD because of its effect in reducing pain crises and the need for transfusions. On the other hand, it has been reported that this medication can precipitate appearance of leg ulcers.^4^^,^^21^ Minniti et al.^4^ are skeptical with relation to this association and report that they did not observe differences between patients with leg ulcers because of SCD who took hydroxyurea in terms of duration of ulcers or response to treatment.^22^ These authors consider that the benefits of hydroxyurea justify its maintenance and only recommend withdrawing the drug if there is a strong suspicion that the substance has contributed to formation of ulcers or if the size of the ulcer has not reduced by at least 50% after 6 months of treatment^4^

## NITRIC OXIDE (NO)

Topical administration of sodium nitrate, a known NO donor, has shown efficacy in preliminary studies^10^ (evidence level B, recommendation level 2). These applications are also related to improved quality of life, especially in young patients.^23^ Random allocation studies with NO-based treatments are needed, since they appear promising if we consider the pathophysiology of sickle-cell ulcers.

## SURGICAL TREATMENT

Grafts are associated with high rates of failure and recurrence.^5^ Certain recommendations exist to attempt to improve the rate of graft success, such as blood transfusions 1 to 2 weeks before surgery and continued for 6 months after the procedure.^24^^,^^25^ Additionally, some surgeons also recommend using anticoagulation with heparin and/or acetylsalicylic acid, antibiotics, and washing grafts in heparinized solutions before applying them.^25^

**Figure 2:**
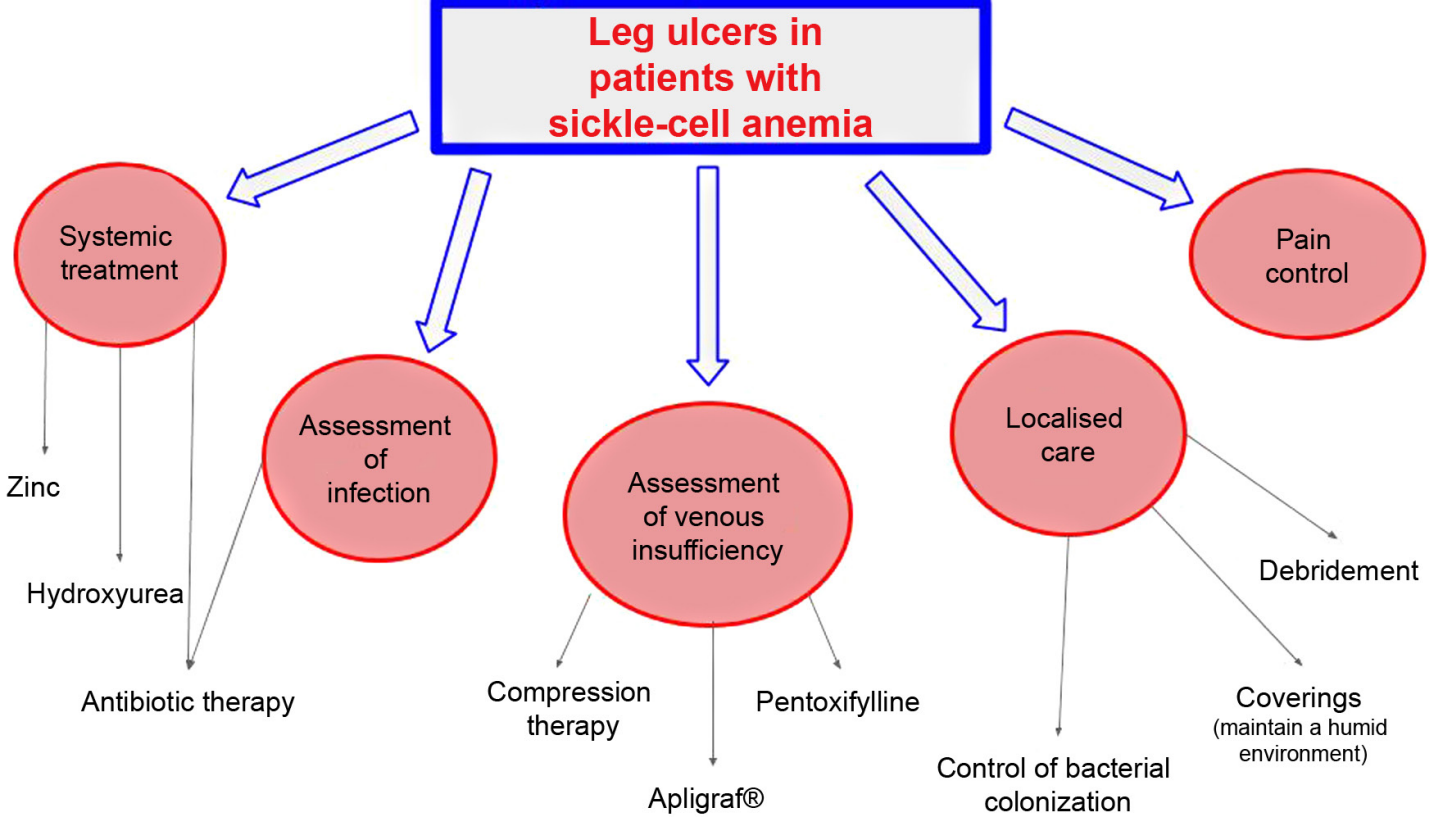
Flow diagram for treatment of leg ulcers in patients with sickle-cell disease, adapted from Altman et al.^5^

Minimally invasive ablation of superficial and perforating axial veins with reflux in patients with CVD and a patent deep vein system is a relatively safe procedure that can accelerate healing and reduce the rate of recurrence when combined with compression therapy.^26^ A detailed assessment of the superficial and deep vein systems and immediate referral to a specialist in angiology and vascular surgery is a critical element of successful treatment for sickle-cell ulcers. Altman et al.^5^ have proposed an algorithm for management of these ulcers in patients with SCD ulcers, which we have adapted (Figure 2).

## PROGNOSIS

Chronic ulcers may be complicated by osteomyelitis, especially when deep, requiring investigation with imaging exams such as bone scintigraphy or magnetic resonance. However, they rarely progress to systemic infection or secondary sepsis.^1^

The classification described towards the start of this article that was proposed by Minniti et al. in 2016^4^ after observation of many patients with SCD and lower limb ulcers enables us to trace a profile of these patients in terms of the time since onset and of relapses.

Thickening of the intima of the common femoral artery beyond 0.9 mm has been correlated with a nine times greater risk of developing leg ulcers in patients with SCD.^27^ Some authors consider that leg ulcers can be taken as an early and visible sign of damage to internal organs, including renal involvement.^28^

## PREVENTION

Prevention is the most important part of management. It consists of avoiding traumas, wearing cotton socks and comfortable footwear, and using insect repellants and emollients, aiming to avoid scaling and itching and prevent scratches.^2^ Secondary prevention consists of instructing patients to visit their physicians in the event of even minimal traumas or injuries.

## CONCLUSIONS

Understanding of the pathophysiology of sickle-cell ulcers, including the role of NO and of venous stasis, has made therapeutic approaches more specific and effective, reducing the time taken to heal and improving the quality of life of patients with SCD.
