# Supplementary material for: Leg ulcers in sickle cell disease patients
Source: J Vasc Bras. 2020 Nov 11;19:e20200054. doi: 10.1590/1677-5449.200054 (PMC8218003; doi:10.1590/1677-5449.200054)
Supplement: Translation

# Translation: Úlceras de perna em pacientes com anemia falciforme

**Authors:** Paula Dadalti Granja, Samuel Braulio Magalhães Quintão, Franciele Perondi, Rosemary Bacellar Ferreira de Lima, Cláudia Labriola de Medeiros Martins, Marcos Arêas Marques, Julio Cesar Peclat de Oliveira

**Affiliations:** 1 Departamento de Medicina Clínica – MMC, Faculdade de Medicina, Universidade Federal Fluminense – UFF, Niterói, RJ, Brasil.; 2 Hospital Universitário Antônio Pedro – HUAP, Niterói, RJ, Brasil.; 3 Hospital Universitário Pedro Ernesto, Universidade do Estado do Rio de Janeiro – UERJ, Rio de Janeiro, RJ, Brasil.; 4 Hospital Universitário Gaffrée e Guinle, Universidade Federal do Estado do Rio de Janeiro – UNIRIO, Rio de Janeiro, RJ, Brasil.; 5 Cirurgia Vascular, Universidade Federal do Rio de Janeiro – UFRJ, Rio de Janeiro, RJ, Brasil.

**Keywords:** anemia falciforme, úlceras, feridas, hemoglobinopatias, complicações

## Abstract

As úlceras de perna são as complicações cutâneas mais comuns em pacientes com anemia falciforme. Acometem principalmente indivíduos homozigotos e são lesões de difícil cicatrização e recidivantes, com impactos físicos, psicológicos e econômicos. Neste trabalho, discutimos a apresentação clínica, o diagnóstico, a fisiopatologia das úlceras falcêmicas e as suas implicações sobre a terapêutica.

## INTRODUÇÃO

A doença falciforme (DF) é um distúrbio genético autossômico recessivo que afeta a forma e a função das hemácias, levando a uma série de complicações sistêmicas. A mutação no cromossoma 11, que codifica a cadeia beta da hemoglobina, resulta na polimerização patológica dessa proteína^1,2^. Como consequência, algumas hemácias se tornam mais densas e perdem sua capacidade de deformar para fluir pela microvascularização, o que implica em vaso-oclusão, inflamação, isquemia, dano tecidual e anemia hemolítica^1,2^. A vasculopatia da DF pode provocar hipertensão pulmonar, crise álgica, síndrome pé-mão, acidente vascular cerebral isquêmico, priapismo e úlcera de membros inferiores^1^.

Até a década de 1960, a DF possuía altas taxas de mortalidade em pacientes jovens, com idade média de dez anos. Os avanços na medicina como o diagnóstico precoce pelo teste de triagem neonatal (“teste do pezinho”), a imunização sistemática, a antibioticoterapia profilática nos cinco primeiros anos de vida, a sistematização da indicação do uso de hemocomponentes e quelantes de ferro, a terapia com hidroxiureia e o rastreamento com Doppler transcraniano para prevenção primária do acidente vascular cerebral têm mudado a história natural dos pacientes com DF^3^. Atualmente, a maioria dos pacientes atinge a idade adulta e, portanto, as complicações da doença se tornam mais frequentes^4^.

As úlceras de perna são as manifestações cutâneas mais comuns na DF^5^ e podem ser incapacitantes^1^. Elas acometem pacientes jovens, a partir da segunda década de vida; são acompanhadas de dor intensa, crônica e contínua; e possuem altas taxas de recorrência. As úlceras afetam significativamente a qualidade de vida, podem estar relacionadas a depressão e acarretam um expressivo aumento nos custos com cuidados de saúde^2,6^.

## METODOLOGIA

O formato deste estudo foi uma revisão não sistemática da literatura a partir da base de dados PubMed, usando os unitermos: *“Sickle cell” and “ulcers”*; *“Sickle cell” and “wounds”*. Metanálises, artigos de revisão publicados nos últimos cinco anos, estudos de alocação aleatória e relatos de caso foram incluídos. Adicionalmente, foram pesquisadas referências dos artigos selecionados.

## EPIDEMIOLOGIA

No Brasil, estima-se que anualmente surjam 3.000 novos casos de DF^7^. As úlceras de perna são dez vezes mais frequentes nos portadores de DF em relação à população geral; portanto, a DF é considerada um forte fator de risco para a ulceração da pele^4^.

A prevalência de feridas em pacientes com DF varia geograficamente, sendo descritos índices que vão desde 75% na Jamaica até 1% na Arábia Saudita^5^. No Brasil, a prevalência dessas úlceras é em 20% dos casos de DF^3^.

Dados jamaicanos indicam que as úlceras de perna são raras antes dos dez anos de idade, ocorrendo mais frequentemente entre 10 e 25 anos. O Estudo Cooperativo de Doença Falciforme nos Estados Unidos estima que a prevalência das úlceras varie entre 5 a 10% e que a incidência máxima ocorra entre 20 e 50 anos^8^.

## FISIOPATOLOGIA

A compreensão dos mecanismos fisiopatológicos é fundamental para uma abordagem terapêutica bem-sucedida. A patogênese da úlcera de perna na DF não é completamente elucidada e parece ser multifatorial. Após a redução dos níveis de oxigênio tecidual, a hemoglobina S polimeriza, se aglomera e distorce a membrana dos glóbulos vermelhos, resultando em hemácias com formato típico de foice. A precipitação intravascular das hemácias resulta em vaso-oclusão, disfunção endotelial, hipercoagulabilidade, inflamação crônica e lesão tecidual isquêmica^5^.

Ocorre hemólise intravascular, que permite que a hemoglobina livre sequestre o óxido nítrico (ON), reduzindo sua ação vasodilatadora sobre o endotélio vascular e, assim, intensificando a vasoconstrição crônica, a hipóxia e a dor^5^. Estudos recentes têm avaliado o papel do ON na DF, sendo observada uma redução dessa substância durante as complicações^9^.

O papel da doença venosa crônica (DVC) na patogênese das úlceras de perna na DF é ressaltado na literatura^5^. O fato de não haver evidências de que os pacientes com DF tenham dificuldade de cicatrização em outras áreas além das pernas sugere que fatores locais específicos estejam relacionados à fisiopatologia dessas feridas^8^. Estudos sobre a mudança no volume de sangue no tornozelo, durante o exercício físico, mostrou tempos de enchimento venoso mais curtos em indivíduos com DF em comparação com controles, sem a doença; e os tempos foram ainda mais reduzidos em pacientes que tinham úlceras falcêmicas em comparação com aqueles que não as possuíam^8^. Logo, a seguinte hipótese foi confirmada: a insuficiência de valvas venosas que drenam a região do tornozelo e a elevação constante da pressão venosa contribuem para a cicatrização lenta e o possível início das úlceras de perna na DF^8^. Estudos de coorte jamaicanos, realizados com Doppler ultrassom portátil, mostraram DVC em 75% de 183 indivíduos com DF em comparação com 39% dos 137 controles sem DF, portanto, com associação significativa (p < 0,001)^8^.

O exame histopatológico evidencia microtrombos e deposição de fibrina na luz de vasos sanguíneos tanto nas úlceras quanto em torno das mesmas, sugerindo hemostasia patológica^10^. A obstrução de microvasos, induzida pela falcização das hemácias, estimula a expressão de elementos de adesão ao endotélio vascular, a agregação plaquetária e a liberação de citocinas pró-inflamatórias, resultando em agravamento da obstrução, exacerbação da isquemia e necrose^5^. Pesquisadores brasileiros detectaram recentemente níveis aumentados de Interleucina-8 em pacientes com DF e úlceras de perna, enfatizando o papel da inflamação na patogênese dessas lesões e sugerindo que essa citocina possa ser considerada como um marcador de mau prognóstico^11^. Também ocorre disfunção autonômica. As respostas reflexas veno-capilares são aberrantes e a vasoconstrição cutânea é mais pronunciada no local da úlcera quando a perna é abaixada^5^.

A combinação de oclusão microvascular, inflamação e trombose, portanto, aumenta o risco de o paciente desenvolver isquemia. O consequente dano tecidual promove eventos cíclicos (valvas danificadas, por exemplo) que agravam ainda mais a lesão tissular e aumentam a retenção de fluidos e a inflamação, promovendo um ambiente que favorece a ulceração^5^.

## CARACTERÍSTICAS CLÍNICAS DA ÚLCERA

Segundo Serjeant et al.^8^, as úlceras de perna na DF podem se iniciar de forma traumática ou espontânea. As lesões traumáticas correspondem a metade dos casos, e as espontâneas surgem dentro da derme. A dor no local da lesão é uma das características mais marcantes das úlceras falcêmicas, que possuem limites bem definidos e bordas elevadas, lembrando perfurações, como observado também em úlceras isquêmicas. Sua base é revestida com tecido de granulação, por vezes recoberto por esfacelo amarelado. Eventualmente, algumas pequenas úlceras aparecem simultaneamente e coalescem, formando uma lesão maior. O edema da extremidade afetada é um achado comum. A pele em torno da ulceração pode ser hipo ou hiperpigmentada, denotando lesões prévias, os folículos pilosos são esparsos e a musculatura, algo atrófica.

Geralmente, as úlceras afetam a pele ao redor dos maléolos medial ou lateral, regiões que possuem maior vulnerabilidade a obstruções mecânicas vasculares, fazendo com que pequenas abrasões se tornem focos de inflamação, isquemia e lesão tecidual^5^. Além disso, o exame físico geralmente revela estigmas de DVC, como hemossiderose cutânea, dermatoesclerose e proeminência das veias superficiais. Outra evidência é a tendência de as úlceras piorarem em posição ortostática e melhorarem com o repouso e com o uso de terapia compressiva^5^.

A cura é lenta e demora meses ou anos. A infecção bacteriana secundária é quase inevitável, e os principais agentes etiológicos são *Staphylococcus aureus*, *Pseudomonas aeruginosa* e *Streptococcus pyogenes.* Por fim, a cicatriz resultante tem uma baixa resistência a tração e a má perfusão devido à vasculopatia cutânea, culminando em maior propensão à reabertura^4^.

## CLASSIFICAÇÃO

Em 2016, Minniti et al.^4^ propuseram três padrões de úlceras de perna em pacientes com anemia falciforme (AF). Essa descrição encontrou suporte de outros estudiosos sobre o tema e foi utilizada em publicações posteriores^2^.

## ÚLCERAS ÚNICAS, ÚLCERAS RECALCITRANTES E ÚLCERAS CRÔNICAS RECORRENTES

A úlcera única é aquela que ocorre em pacientes que apresentam apenas um episódio de úlcera durante a vida, com cicatrização em alguns meses. Tipicamente acontece na segunda década de vida e pode recidivar em caso de estresse. Esses pacientes têm baixa frequência de crises de dor e podem ter complicações pulmonares ou renais.

Já as úlceras recalcitrantes são pequenas úlceras que recorrem a cada 6 a 12 meses por vários anos. São observadas em um quarto dos pacientes e costumam responder melhor à terapêutica. Embora os indivíduos tenham muito receio de apresentar recorrências, geralmente não costumam ficar gravemente debilitados pelas lesões.

Por fim, as úlceras crônicas recorrentes são lesões que persistem por anos ou recidivam no mesmo local ou nas proximidades. Essa forma de apresentação causa a dor mais debilitante e crônica, e o paciente sofre com depressão, incapacidade e desemprego. Embora haja cicatrização em 75 a 80% dos casos, muitos pacientes podem apresentar úlceras que duram por mais de 20 anos ou que nunca cicatrizam. Em alguns casos, a amputação do membro pode ser considerada para melhorar a qualidade de vida.

## DIAGNÓSTICO

Embora a maior parte dos pacientes com AF tenha diagnóstico precoce em grandes centros, devemos estar atentos a essa possibilidade em pacientes jovens com úlceras de perna, pois eventualmente o diagnóstico da DF pode ocorrer tardiamente, pela complicação.

A história deve ser detalhada, incluindo informações sobre o tratamento atual e as complicações de tratamento anteriores. Ao exame físico, podemos avaliar se há hipo ou hiperpigmentação perilesional, edema do membro, linfonodos inguinais e o tamanho da úlcera. Devemos observar também as características e o volume do exsudato e o aspecto do leito. O diagnóstico diferencial das úlceras em pacientes com DF inclui as inúmeras causas de úlceras crônicas de perna, como: as úlceras vasculares (arterial, venosa e mista), a úlcera hipertensiva (Martorell), as úlceras infecciosas (no nosso meio, destacam-se leishmaniose, esporotricose e micobacterioses), as úlceras induzidas por medicamentos (hidroxiureia e metotrexate, por exemplo), as úlceras relacionadas a neoplasias (como carcinoma basocelular, espinocelular, melanoma e metástases cutâneas), as úlceras relacionadas a doenças autoimunes (esclerose sistêmica, lupus eritematoso sistêmico, artrite reumatoide), além de condições cutâneas primárias (necrobiose lipoídica, sarcoidose, pioderma gangrenoso, por exemplo)^12^. O diagnóstico laboratorial da DF busca identificar a hemoglobina S (HBS) ou outras hemoglobinas variantes e deve ser feito através da eletroforese da hemoglobina.

Testes laboratoriais como urinálise, hemograma e bioquímica devem ser solicitados. Podem ser observados microalbuminúria e marcadores de hemólise crônica grave, que auxiliam no diagnóstico^2^.

O exame histopatológico de biópsia da úlcera pode revelar margens epiteliais atróficas, aumento da vascularidade, vasculopatia com oclusão vascular, processo inflamatório crônico, microtrombos e deposição de fibrina na íntima^2^. Embora esses achados não sejam muito específicos, são particularmente úteis no diagnóstico diferencial com outras causas de úlceras de membros inferiores.

## TRATAMENTO

O tratamento das úlceras de perna no paciente com DF deve considerar uma abordagem holística para redução dos impactos físicos e psíquicos decorrentes de uma enfermidade crônica, com lento processo de cicatrização e altas taxas de recorrência. No entanto, são escassas as diretrizes, baseadas em evidências científicas, que orientem condutas de tratamento adequadas. Assim, os profissionais de saúde baseiam-se em revisões críticas da literatura e na experiência pessoal e clínica para a definição das condutas de tratamento das úlceras^4^.

## CUIDADOS GERAIS

No paciente com feridas, a adesão ao tratamento pode ser considerada o principal fator determinante da cicatrização bem-sucedida^5^. Uma abordagem multidisciplinar é essencial, com acompanhamento próximo de hematologista, cirurgião vascular, angiologista, dermatologista, enfermeiro, nutricionista e psicólogo.

Do ponto de vista nutricional, devemos ficar atentos à possibilidade de deficiência de zinco, cuja correção pode facilitar a cicatrização. A recomendação atual é de 220 mg de sulfato de zinco três vezes ao dia, com reavaliação após 3 a 4 semanas e suspensão caso os valores de referência tenham sido alcançados^5^. A pesquisa e o tratamento da trombose venosa superficial e/ou profunda são fundamentais, eventualmente com necessidade de anticoagulação.

O manejo da dor deve ser uma prioridade. Por conta do perfil de efeitos adversos do uso crônico de opioides, geralmente são indicados anti-inflamatórios não esteroides. Altman et al. sugerem, a partir de observações pessoais, o bloqueio nervoso regional, com a vantagem de oferecer vasodilatação secundária pela redução de catecolaminas liberadas pelo estresse^5^. Por outro lado, a adesão pode ser comprometida por necessidade de procedimento invasivo, risco de infecção de tecidos moles e necessidade de visitas frequentes para reposição.

## CUIDADOS LOCAIS

A cicatrização de feridas é um processo complexo e dinâmico que pode ser simplificado em três fases. Na primeira fase, inflamatória, ocorre extravasamento de sangue e as plaquetas liberam fatores de crescimento que atraem e ativam fibroblastos, macrófagos e leucócitos. Durante essa fase, o foco terapêutico deve ser no desbridamento e controle de infecção. A maioria das feridas crônicas encontra-se nessa fase. Quanto mais tempo a ferida ficar na fase inflamatória, menor deposição de colágeno haverá, resultando em menor resistência à tração da nova pele. Na segunda fase, proliferativa, inicia-se a epitelização, forma-se o tecido de granulação e ocorre a contração da ferida. Na última fase, de maturação e remodelamento, ocorre degradação do colágeno controlado por enzimas proteolíticas^4^.

Os princípios básicos dos tratamentos de feridas têm sido didaticamente simplificados pela sigla TIME: debridamento Tecidual; controle da Infecção/Inflamação; Manutenção/equilíbrio do ambiente úmido (*Moisture*); e Epitelização das bordas da lesão^13^. O tratamento do leito da úlcera é fundamental no processo de cicatrização. O debridamento do biofilme, da fibrina e do tecido necrótico não viável na base e nas bordas é essencial, sendo considerado o primeiro passo para remover as barreiras físicas que interferem com a cicatrização^14^. O método (autolítico, enzimático, biológico, mecânico ou cirúrgico) será escolhido a partir do tipo de ferida, da localização anatômica, da extensão e da disponibilidade. Por conta da dor, o debridamento cirúrgico pode necessitar de analgesia ou até mesmo anestesia.

O controle da colonização bacteriana é importante na escolha da cobertura, e algumas substâncias, como prata, iodine e polihexametileno biguanida, têm propriedades antibacterianas e podem reduzir a colonização crítica^13,15^. Não há evidências de que tratar úlceras clinicamente não infectadas com antibióticos sistêmicos previna infecção ou melhore a cicatrização. Quando houver sinais clínicos de infecção, uma amostra profunda de tecidos moles ou osso pode ser colhida após o debridamento, para cultura e avaliação da sensibilidade a antimicrobianos.

A manutenção do ambiente úmido pode ser obtida com a escolha da cobertura adequada, como hidrocoloides, hidrogéis, alginatos, colágeno ou substitutos biológicos de pele. Algumas dessas coberturas, inclusive, oferecem propriedades anti-inflamatórias e de debridamento autolítico.

Uma matriz de peptídeos, conhecida como RDG, foi mencionada na revisão Cochrane (2014) como eficaz na redução do tamanho das úlceras tratadas, em comparação com o placebo^16^. No entanto, as evidências não são muito consistentes. Outros ensaios avaliados nessa metanálise tiveram alto risco de viés e falharam ao mostrar efeitos benéficos^17^.

Novas tecnologias, como o laser de baixa intensidade (LBI), têm sido utilizadas com o objetivo de acelerar a cicatrização, melhorando a qualidade de vida dos pacientes^5,18^ (nível de evidência 4, relato de caso). Um efeito fundamental é sua capacidade de reduzir a dor, o que permite uma abordagem mais efetiva durante o curativo.

## TRATAMENTO DA DVC

A terapia compressiva é encorajada para prevenção e tratamento do edema, especialmente quando são observados sinais clínicos de DVC. As meias elásticas de compressão graduada são úteis para a prevenção, enquanto as bandagens de multicamadas são recomendadas para o tratamento. Uma alternativa são as bandagens autoaplicáveis e autoajustáveis com velcro, embora mais sujeitas a erros por parte do aplicador^5^. O grau recomendado de compressão para acelerar a cicatrização em pacientes com DVC é 1 (nível de evidência A) e, na redução do risco de recorrência, grau de recomendação 2 (nível de evidência B)^5^. Ilustramos a resposta da terapia compressiva com bota de Unna em paciente acompanhado em nosso ambulatório (Figura 1).

**Figura 1:** Paciente de 39 anos, diagnosticado com doença falciforme na infância. Nas imagens, lesão ulcerada medindo cerca de 8 cm em seu maior diâmetro, com leito coberto por fibrina na maior parte da sua extensão (A). Feita abordagem com poliaminopropil biguanida seguida pela aplicação de cobertura a base de carboximetilcelulose sódica e prata além da colocação de bota de Unna semanalmente (B). Paciente evoluiu com cicatrização completa após 2 meses de tratamento (C).

A pentoxifilina, um derivado da metilxantina, é um inibidor competitivo da fosfodiesterase que mostrou propriedades antioxidantes e redutoras da inflamação. Além disso, reduz a viscosidade do sangue, o potencial de agregação plaquetária e a formação de coágulo^19^. O seu uso em úlceras falcêmicas já havia sido proposto em 1990, justificado por seus efeitos redutores da falcização das hemácias *in vitro* e resolução de crises vaso-oclusivas na AF (nível de evidência C, grau de recomendação 4)^20^.

Essa droga foi fortemente recomendada para tratamento de úlceras de estase venosa (nível de evidência B, grau de recomendação 1) e alguns autores consideram que também deva ser utilizada em pacientes com úlceras falcêmicas pela alta frequência de DVC observada nesses casos5. O mesmo grupo5 também sugere que seja utilizado um substituto biológico de pele (Apligraf®, Organogenesis Inc., Canton, Massachusetts), composto por queratinócitos e fibroblastos cultivados e colocados sobre uma matriz de colágeno, no tratamento de úlceras de perna em pacientes com AF e DVC, mas que não tenham respondido às terapêuticas convencionais em 4 a 6 semanas. No caso de úlceras por DVC, o nível de evidência é A, grau 2^5^.

## HIDROXIUREIA

A hidroxiureia é amplamente utilizada no tratamento da AF pelo seu efeito em reduzir as crises de dor e a necessidade de transfusão. Por outro lado, existe a descrição de que esse medicamento possa precipitar o aparecimento de úlceras de perna^4,21^. Minniti et al.^4^ se mostram céticos em relação a essa associação e relatam não terem observado diferença entre os pacientes com úlceras de perna por AF que usavam hidroxiureia na duração das úlceras e na resposta ao tratamento^22^. Tais autores consideram que os benefícios da hidroxiureia justificam a sua manutenção e só recomendam a suspensão da droga caso haja forte suspeição de que a substância tenha contribuído para a formação da úlcera ou caso não haja redução de pelo menos 50% do tamanho da úlcera em 6 meses de tratamento^4^

## ÓXIDO NÍTRICO (ON)

A aplicação tópica de nitrato de sódio, um conhecido doador de ON, mostrou eficácia em estudos preliminares^10^ (nível de evidência B, grau de recomendação 2). Essas aplicações também estão relacionadas a uma melhora na qualidade de vida, especialmente em indivíduos mais jovens^23^. Estudos de alocação aleatória com terapias baseadas em ON precisam ser realizados e parecem promissores quando levamos em conta a fisiopatologia das úlceras falcêmicas.

## TRATAMENTO CIRÚRGICO

Os enxertos são relacionados a altas taxas de insucesso e recorrência^5^. Algumas recomendações são feitas para tentar melhorar a taxa de adesão dos enxertos, como transfusões sanguíneas 1 a 2 semanas antes da cirurgia e continuando por 6 meses após o procedimento^24,25^. Adicionalmente, alguns cirurgiões recomendam o uso de anticoagulação com heparina e/ou ácido acetilsalicílico (AAS), antibióticos e lavagem dos enxertos em solução heparinizada antes da sua aplicação^25^.

A ablação minimamente invasiva de veias axiais superficiais e perfurantes com refluxo em pacientes com DVC e sistema venoso profundo pérvio é um procedimento relativamente seguro que leva à cicatrização mais rápida e à redução da taxa de recorrência quando combinado com terapia compressiva^26^. Uma avaliação minuciosa do sistema venoso superficial e profundo e a referência imediata a um especialista em angiologia e cirurgia vascular é um componente crítico do tratamento bem-sucedido das úlceras falcêmicas. Altman et al.^5^ inclusive propuseram um algoritmo para abordar as úlceras nos pacientes com úlceras por AF, que adaptamos (Figura 2).

**Figura 2:** Fluxograma de tratamento de úlceras de perna em pacientes com anemia falciforme, traduzido e adaptado de Altman et al.^5^

## PROGNÓSTICO

As úlceras crônicas podem complicar com osteomielite, especialmente quando profundas, necessitando de investigação com exames de imagem como cintilografia óssea ou ressonância magnética. No entanto, raramente evoluem para infecção sistêmica ou sepse secundária^1^.

A classificação descrita anteriormente, proposta por Minniti et al. em 2016^4^, após observação de muitos pacientes com AF e úlceras de membros inferiores, nos permite também traçar um perfil desses pacientes em relação ao tempo de evolução e recidivas.

O espessamento da íntima da artéria femoral comum, acima de 0,9 mm, foi correlacionado com risco nove vezes maior de desenvolver úlceras de perna em pacientes com AF^27^. Alguns autores consideram que as úlceras de perna possam ser encaradas como sinal precoce e visível de lesões de órgãos internos, incluindo acometimento renal^28^.

## PREVENÇÃO

A prevenção é a parte principal da abordagem. Consiste em evitar trauma, usar meias de algodão, sapatos confortáveis, repelentes contra insetos e emolientes, buscando evitar descamação e prurido, e prevenir escoriações^2^. A prevenção secundária consiste em orientar os pacientes a procurarem seu médico em caso de traumas ou lesões mínimas.

## CONCLUSÃO

A compreensão da fisiopatologia da úlcera falcêmica, como o papel do ON e da estase venosa, tem tornado as abordagens terapêuticas mais específicas e eficazes, reduzindo os tempos de cicatrização e melhorando a qualidade de vida do paciente com DF.
